# BMP9 Protects Septal Neurons from Axotomy-Evoked Loss of Cholinergic Phenotype

**DOI:** 10.1371/journal.pone.0021166

**Published:** 2011-06-13

**Authors:** Ignacio Lopez-Coviella, Tiffany J. Mellott, Aletta C. Schnitzler, Jan K. Blusztajn

**Affiliations:** 1 Department of Psychiatry, Boston University School of Medicine, Boston, Massachusetts, United States of America; 2 Department of Pathology and Laboratory Medicine, Boston University School of Medicine, Boston, Massachusetts, United States of America; Nathan Kline Institute and New York University School of Medicine, United States of America

## Abstract

**Background:**

Cholinergic projection from the septum to the hippocampus is crucial for normal cognitive function and degeneration of cells and nerve fibers within the septohippocampal pathway contributes to the pathophysiology of Alzheimer's disease. Bone morphogenetic protein (BMP) 9 is a cholinergic differentiating factor during development both *in vivo* and *in vitro*.

**Methodology/Principal Findings:**

To determine whether BMP9 could protect the adult cholinergic septohippocampal pathway from axotomy-evoked loss of the cholinergic phenotype, we performed unilateral fimbria-fornix transection in mice and treated them with a continuous intracerebroventricular infusion of BMP9 for six days. The number of choline acetyltransferase (CHAT)-positive cells was reduced by 50% in the medial septal nucleus ipsilateral to the lesion as compared to the intact, contralateral side, and BMP9 infusion prevented this loss in a dose-dependent manner. Moreover, BMP9 prevented most of the decline of hippocampal acetylcholine levels ipsilateral to the lesion, and markedly increased CHAT, choline transporter CHT, NGF receptors p75 (NGFR-p75) and TrkA (NTRK1), and NGF protein content in both the lesioned and unlesioned hippocampi. In addition, BMP9 infusion reduced bilaterally hippocampal levels of basic FGF (FGF2) protein.

**Conclusions/Significance:**

These data indicate that BMP9 administration can prevent lesion-evoked impairment of the cholinergic septohippocampal neurons in adult mice and, by inducing NGF, establishes a trophic environment for these cells.

## Introduction

The cholinergic neurons of the medial septal nucleus send their axons to the hippocampus providing it with a robust modulatory input mediated by their neurotransmitter, acetylcholine (ACh). Although not numerous (approximately 3000 in the mouse) [1], their significance includes critical functions in processes of learning, memory, attention, and sleep [2], [3]. Degeneration and/or malfunction of the septohippocampal cholinergic system is thought to contribute to cognitive decline of old age and to memory loss characteristic of Alzheimer's disease [4]. Lesions of this pathway in laboratory animals impair learning and memory in various tests [5]. Because these abnormalities are related to the compromised cholinergic neurotransmission and since the septohippocampal pathway is the main extrinsic source of ACh to the hippocampus, multiple experimental strategies aimed at restoring the content of this neurotransmitter in the hippocampus have been tried. For example, grafts of ACh-producing cells ameliorated some lesion-evoked cognitive deficits [6], [7]. In an effort to correct similar deficits in AD, a number of therapeutic approaches have also been undertaken to increase the levels of brain ACh in humans. Some of these strategies are based on the use of acetylcholinesterase inhibitors, to reduce the rate of degradation of ACh in brain [8]. Other approaches include the use of growth factors known to be neurotrophic for cholinergic neurons and to be effective in animal models – NGF being the best characterized [9]–[17] –and clinical trials on NGF gene therapy in an attempt to increase the functional state and survival of cholinergic neurons in AD patients have been initiated [18].

We have previously shown that bone morphogenetic protein (BMP) 9 is an inducer of the cholinergic phenotype of septal neurons in culture and in embryonic brain *in vivo* and a maintenance factor for these cells [19]. Moreover, in fetal septal cell cultures, BMP9 induces the expression of multiple genes, a large fraction of which belong to the transcriptome of the septal cholinergic neurons [20], indicating that BMP signaling may participate in the maturation of these cells more broadly, i.e. beyond their neurotransmitter specification. Interestingly, several BMP9-induced genes encode proteins with trophic activities for cholinergic neurons, suggesting that BMP signaling helps to generate a favorable milieu for these cells. Indeed, we have recently demonstrated that some of the effects of BMP9 are apparently mediated by autocrine action of NGF that is induced in these neurons in response to BMP9 [21]. Multiple BMPs and their antagonists also function in controlling hippocampal development and modulate adult neurogenesis that is characteristic to this brain region [22]. In the present study we found that intracerebroventricular (ICV) infusion of BMP9 prevents the loss of cholinergic neurons, as defined by their expression of the ACh-synthesizing enzyme choline acetyltransferase (CHAT), in the septum following unilateral fimbria-fornix transection in mice and upregulates the expression of hippocampal NGF and its receptors NGFR-p75 and TRKA (NTRK1).

## Results

### BMP9 prevents lesion-evoked loss of choline acetyltransferase (CHAT) positive medial septum neurons

**Figure 1:**
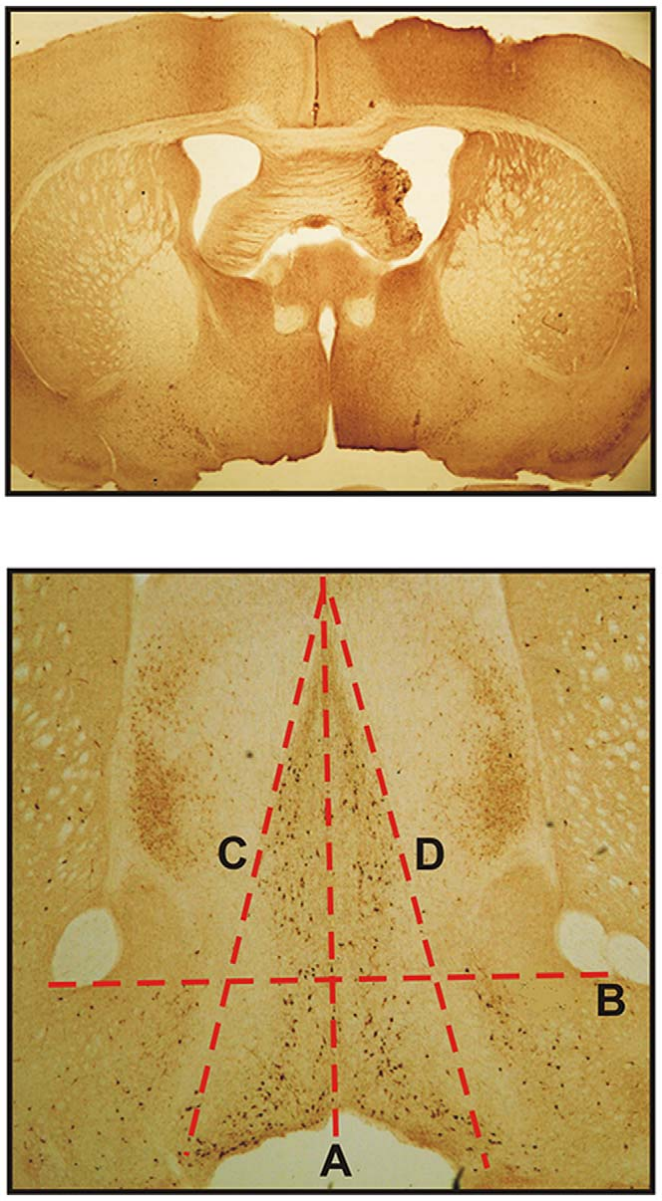
Sections of mouse brain showing a representative image of the unilateral transection of fimbria-fornix (top panel) and the ROI stained with an anti-CHAT antibody (Bottom Panel). CHAT-positive cells are seen in the medial septum, the diagonal band and in the striatum (bottom panel). The lines show the ROI used for cell count analysis in the medial septum. The ROI was defined by a triangular shape that extended, dorso-ventrally, from the apex of the medial septum (A) to an imaginary line connecting the lower limits of the anterior commissures on each hemisphere (B) and, medio-laterally, from the midline (A) to the outer limits of the medial septal area (C and D).

**Figure 2:**
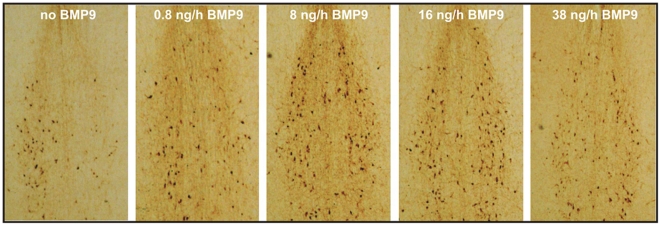
Representative photomicrographs of anti-CHAT antibody stained basal forebrain areas of mice with a unilateral septohippocampal lesion (right side of the images) following six days of constant intracerebroventricular infusion of vehicle (no BMP9) or BMP9 at doses indicated. Note a dramatic reduction of the number of CHAT-positive cells in the control brain and sparing of these neurons when BMP9 was administered.

**Figure 3:**
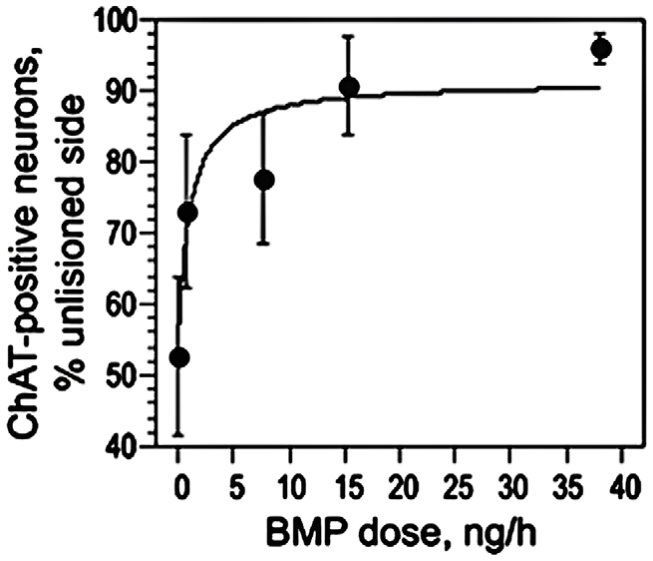
BMP9 protects medial septum cholinergic neurons from axotomy-evoked degeneration. CHAT-positive cells were counted as shown in Fig. 1 on both sides of the medial septum and the average cell number on the lesioned side is expressed as % of the average cell number on the intact side. See Fig. 2 for representative sections. Using the best fit to a rectangular hyperbola (R^2^ = 0.89) the EC_50_ value for BMP9 was 1 ng/h. Note, at 15 ng/h and 38 ng/h BMP9 prevented all loss of CHAT-positive neurons.

To assess the *in vivo* activity of BMP9 on injured adult septal cholinergic neurons, we performed unilateral fimbria-fornix transections in mice and treated them with varying doses of BMP9 administered via continuous ICV infusion over a 6-day period. Immunohistochemical staining of the septum with an antibody for CHAT was then used to determine the effects of the lesion and of BMP9. Serial images of the septal/diagonal band area from CHAT-immunostained histological sections were processed for cell number quantification within the region of interest (ROI) encompassing the individual left and right medial septal nuclei defined by triangular shapes shown in Figure 1 as described in the methods. The ROI were analyzed for cell number in at least three non-consecutive sections per animal. As expected, multiple CHAT-immunoreactive cells within the medial septum and the diagonal band were observed. Mice subjected to the fimbria-fornix transection and infused with PBS alone, were characterized by a 50% loss in the number of the CHAT-positive cells in the medial septum ipsilateral to the lesion (Fig. 2 and 3). Sparing of the cholinergic neurons was evident with increasing doses of BMP9, and there was little or no loss of these neurons in animals receiving 15 and 38 ng/h of BMP9 (Figs. 2 and 3), indicating that BMP9 was highly effective in preventing the loss of CHAT-positive neurons whose axons had been transected. The dose-dependent and saturable action of BMP9 could be described by a rectangular hyperbola with a good fit (R^2^ = 0.89) and permitted the estimation of the EC_50_ value for BMP9 at 1 ng/h (Fig. 3).

### BMP9 attenuates lesion-evoked reduction of acetylcholine levels in the hippocampus

**Figure 4:**
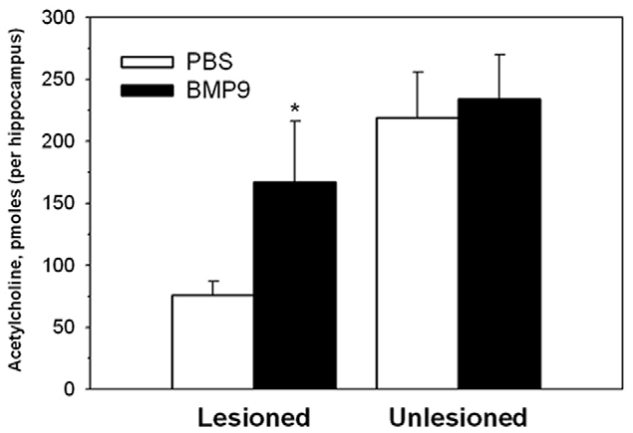
Acetylcholine content in hippocampus measured by HPLC in mice with a unilateral septohippocampal lesion following seven days of constant intracerebroventricular infusion of vehicle or 8 ng/h of BMP9. Note a significant (*p<0.01; 72%) sparing of the loss of hippocampal ACh content in lesioned mice receiving BMP9.

To determine whether BMP9 could affect ACh levels in the hippocampus, we performed similar experiments in mice with fimbria-fornix transections using a small, yet close to a fully effective dose of BMP9 identified above (8 ng/h, via a 6-day ICV infusion; Fig. 3), and measured ACh content in both lesioned and unlesioned hippocampi. Seven days after the lesion, ACh content in the hippocampus ipsilateral to the lesion was reduced to 34% compared to the unlesioned side in mice that had been infused with PBS, whereas ACh levels in the hippocampus ipsilateral to the lesion in mice infused with BMP9 were reduced to only 72% (Fig. 4). Thus, BMP9 treatment significantly (p<0.01) prevented the reduction in ACh levels in the lesioned hippocampus when compared to untreated animals. The magnitude of this effect was the same regardless of whether ACh content was expressed per hippocampus, or per mg of protein.

### BMP9 increases cholinergic marker expression in the hippocampus

**Figure 5:**
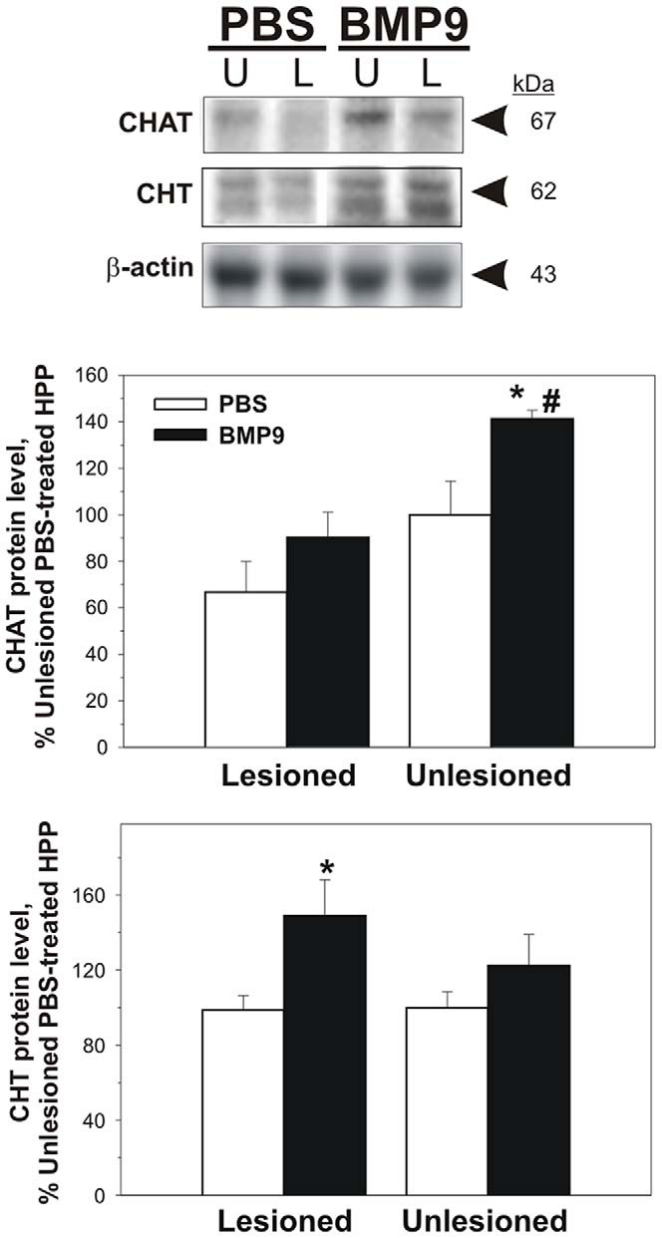
Protein levels of cholinergic markers in hippocampus (HPP). CHAT and CHT were measured by Western blot (top panel; representative blot images) in hippocampi from lesioned and unlesioned side, the intensities of the immunoreactive bands were quantified, normalized to those of beta actin and the data are expressed as % of the values from PBS-infused mice on the unlesioned side. Two-way ANOVA of these data for CHAT revealed a statistically significant effect of the lesion (p = 0.002) and of BMP9 (p = 0.01). The post hoc test revealed that the reduction of the CHAT levels in the PBS-treated mice on the lesioned side was close to statistical significance (p = 0.054) and that BMP9 increased CHAT levels on the intact side (p = 0.02). Note, that the comparison of the hippocampal CHAT levels between the control animals (PBS-infused unlesioned side) and the BMP9-infused lesioned side showed no significant difference (p = 0.54) indicating that the BMP9 treatment tended to cause normalization of the CHAT protein levels. (* p<0.05 vs. PBS; # p<0.05, vs. lesioned BMP9-treated). Two-way ANOVA of the data for CHT revealed an effect of BMP9 (p<0.02); (* p<0.02 BMP9 vs PBS on the lesioned side).

To determine whether cholinergic cell marker expression was modulated in the hippocampus of BMP9 treated mice with fimbria-fornix lesions, we used immunoblotting to measure protein levels of CHAT and choline transporter CHT (a protein that catalyzes the uptake of choline used for ACh synthesis in cholinergic neurons). CHAT protein levels were reduced with the lesion in both BMP9-treated and untreated mice (Fig. 5). BMP9 increased CHAT levels in both lesioned and unlesioned hippocampi. However, these levels were only significantly (p<0.05) different in the unlesioned hippocampus. Two-way ANOVA revealed a statistically significant effect of the lesion (p = 0.002) and of BMP9 (p = 0.01). The post hoc test revealed that the reduction of the CHAT levels in the PBS-treated mice on the lesioned side was close to statistical significance (p = 0.054) and that BMP9 increased CHAT levels on the intact side (p = 0.02). Note, that the comparison of the hippocampal CHAT levels between the control animals (PBS-infused unlesioned side) and the BMP9-infused lesioned side showed no significant difference (p = 0.54) indicating that the BMP9 treatment tended to cause normalization of the CHAT protein levels. CHT protein levels were elevated in the hippocampus of BMP9 treated mice compared to PBS-infused controls, but only those in the lesioned side were statistically different from controls (p<0.05; Fig. 5).

### BMP9 increases NGF and NGF-receptors levels in the hippocampus

**Figure 6:**
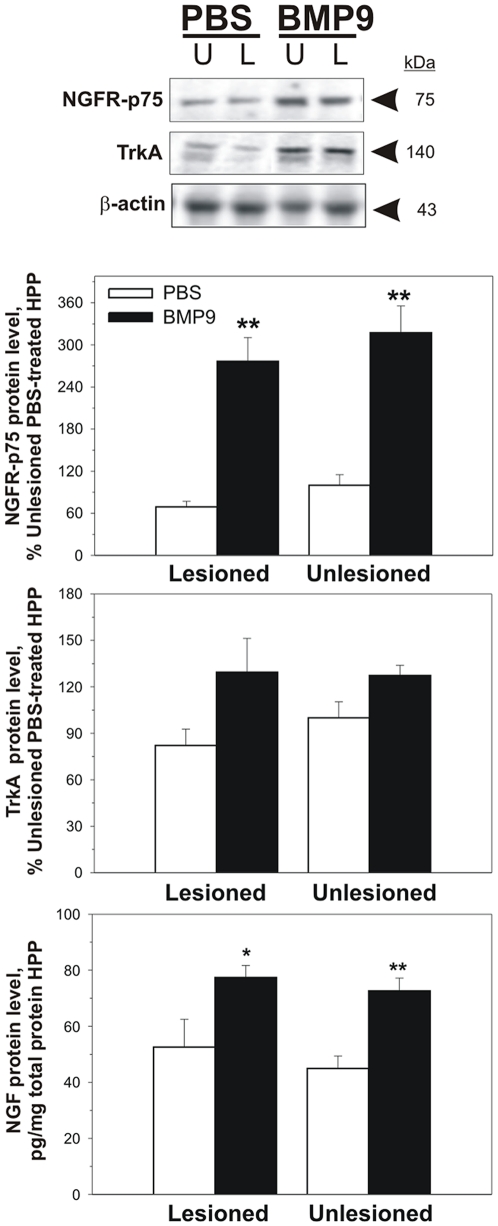
NGFR-p75, TrkA and NGF protein levels in hippocampus (HPP). Mice were treated as in Fig. 4. NGFR-p75 (** p<0.01 BMP vs PBS on the same side) and TrkA (significant treatment effect of BMP9 by two-way ANOVA, p<0.05) were measured by Western blot (top panel) and the data quantified as in Fig. 5. NGF was measured by ELISA (* p<0.05, ** p<0.01 BMP9 vs PBS on the same side).

It is well established that basal forebrain cholinergic neurons benefit from hippocampal NGF as a trophic factor for survival and maintenance of their cholinergic phenotype [9]–[12]. We have recently shown that BMP9 can induce NGF as an autocrine/paracrine cholinergic trophic factor in developing basal forebrain neurons [21]. To determine whether BMP9 had an effect on NGF content in the hippocampus of fimbria-fornix lesioned mice and, perhaps, explain some of our data described above, we analyzed the levels of NGF protein in the hippocampus of these mice by ELISA. NGF levels were increased in the hippocampus of BMP9 treated mice by 47% and 61% on the lesioned and unlesioned side, respectively, compared to the corresponding levels in the hippocampus of PBS infused control animals (Fig. 6).

To determine whether BMP9 had an effect on NGF receptors levels, we measured NGFR-p75 and TRKA (NTRK1) by Western blot. NGFR-p75 protein levels were 4.3-fold and over 2.5-fold higher in the hippocampus of lesioned and unlesioned BMP9 treated mice, respectively, compared to PBS-infused animals (Fig. 6). BMP9 also increased the levels of TrkA both in lesioned and unlesioned hippocampi (overall significant treatment effect, p<0.05, by two-way ANOVA; Fig. 6).

### BMP9 decreases basic-FGF content in the hippocampus

**Figure 7:**
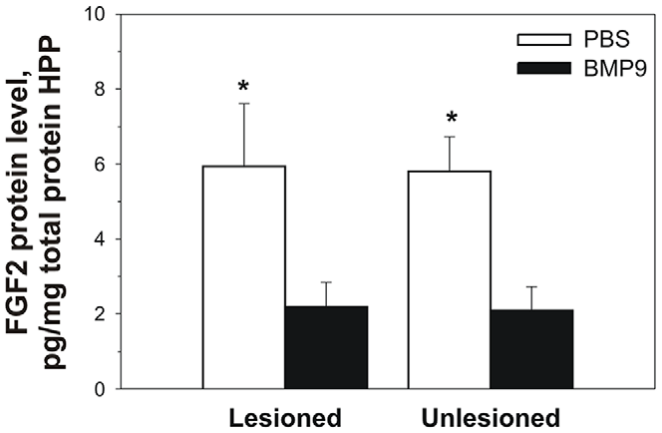
FGF2 protein levels in hippocampus (HPP). Mice were treated as in Fig. 4. FGF2 was measured by ELISA (* p<0.05 BMP9 vs PBS on the same side).

The interaction between BMP and other growth factors promoting progenitor cell proliferation, such as basic FGF (FGF2), is essential during brain development and it may also be important in cell repair following brain injury. To determine whether BMP9 affects FGF2 content in the hippocampus, we measured the levels of this growth factor by ELISA. BMP9 significantly reduced FGF2 levels in both lesioned and unlesioned hippocampi (p<0.05), whereas the levels of this growth factor were not significantly different in vehicle treated mice regardless of the lesion (Fig. 7).

## Discussion

These data show that BMP9 administered at the time of a septo-hippocampal transection prevents the loss of septal cholinergic neurons (i.e. CHAT-immunopositive cells) normally observed with this type of injury. They also show that BMP9 partially prevents the loss of ACh and other cholinergic markers in the lesioned hippocampus, and that BMP9 induces hippocampal NGF that could in part be responsible in mediating the above effects.

Unilateral fimbria-fornix transection has been one of the most successful models in the study of the effects of growth factors on injured septal cholinergic neurons. Following this procedure, the ipsilateral septal neurons show downregulated expression of CHAT [13]–[17] and of another cholinergic marker the vesicular ACh transporter [23]. It is generally thought that following fimbria fornix axotomy the cholinergic septal neurons tend not to degenerate and die [24], [25], instead, devoid of contact with their targets these neurons lose their cholinergic phenotype without dying [12]. Whether the actions of BMP9 observed here result from a direct effect of BMP9 on the cholinergic neurons subjected to the lesion, or indirectly mediated by other factors, or both, remains uncertain. It has been shown that, following-fimbria fornix transection, NGF can rescue septal neurons from the loss of their cholinergic phenotype when administered at the time of the lesion [14], as is the case for BMP9 in the present study. Thus, our data from this and previous studies [21] suggest that BMP9 bears similarity to the actions of NGF in a variety of ways including increased ACh production and CHAT expression. As noted above we found that BMP9 can induce the synthesis and release of NGF in septal cultures, and that NGF can partially mediate some of the effects of BMP9 [21]. Moreover, like NGF, BMP9 can increase gene expression of NGF receptor NGFR-p75 [21], [26]. NGFR-p75 would be expected to potentiate NGF signaling via the NGF signaling receptor, TrkA [27] whose hippocampal expression is also increased in BMP9-infused mice. Thus, in theory, BMP9 by increasing both NGF protein synthesis and, concurrently, upregulating NGF receptor (NGFR-p75 and TrkA) expression in septal neurons, could rescue their cholinergic phenotype upon delayed administration following fimbria-fornix lesions. Although we did not explore this possibility, this could have direct translational implications in the search for treatments of disease states affecting basal forebrain cholinergic neurons with dysfunction of NGF and its receptors, such as Alzheimer's disease [4], [28].

BMP signaling plays an important role in both neurogenesis and gliogenesis [29]. The interaction between BMP and other growth factors, such as FGF2, is central in the maturation of the nervous system [30] and could also, play a significant role during neuronal cell repair following brain injury. We found that BMP9 significantly reduced FGF2 levels in both lesioned and unlesioned hippocampi, whereas the levels of this growth factor remained unchanged in vehicle treated mice regardless of the lesion. FGF2 promotes the proliferation of progenitor cells, preventing their exit from the cell cycle [31]. BMPs, in contrast, tend to reduce cell proliferation, promoting cell differentiation and cell lineage restriction. During development, these apparently opposing effects may contribute to a specific cell fate [32]. Our data suggest that, following fimbria-fornix transection, BMP9 may indirectly support and maintain the cholinergic phenotype by an alternative mechanism, namely, by reducing FGF levels and maintaining the state of cholinergic differentiation.

In spite of an apparently complete, microscopically verified, transection of fimbria fornix, detectable levels of ACh still persisted 6-days post-lesion in the hippocampus, albeit drastically reduced, and treatment with BMP9 partially blocked this loss of hippocampal ACh content. The most likely explanation for this is apparent upregulation of ACh synthesis and/or sprouting of the nerve terminals of the septal cholinergic neurons that project to the hippocampus using the supracallosal pathway [33]–[35] and a ventral pathway (ansa lenticularis) [36], [37]. In addition, early studies have pointed out the possible presence of intrinsic cholinergic neurons in the hippocampus [38]–[40] and we [41] and others [42] have observed CHT-immunoreactive cells in the rat hilus. Note that in the current study we found a significant increase in CHT protein content evoked by BMP9 in the lesioned hippocampus, but not the unlesioned side. CHT is responsible for the high affinity transport of choline into synaptic nerve terminals and can be the rate limiting step in ACh synthesis [3]. Previous mouse model studies (e.g. CHAT haploinsufficiency [40]) have shown that upregulation of CHT expression can provide functional compensation in states of cholinergic dysfunction [43]. In our lesion experiments, the loss of septohippocampal input to the lesioned hippocampus could render intrahippocampal cholinergic neurons more susceptible to BMP9, and upregulate CHT protein content to compensate for the loss of extrinsic cholinergic innervation. In addition, our results show that BMP9 significantly increased CHAT protein content in the unlesioned hippocampi indicating that it increases CHAT expression in adult basal forebrain cholinergic neurons as it does during development [19]. In the lesioned hippocampi treated with BMP9 CHAT protein levels were not statistically different than those in PBS-treated intact and lesioned controls, suggesting that BMP9 treatment tended to reduce the lesion-evoked loss of the hippocampal CHAT.

In conclusion our data show that intracerebral administration of BMP9 protects the axotomized septal cholinergic neurons from the loss of their neurotransmitter phenotype and increases hippocampal expression of NGF, a critical growth factor for these neurons. Together the results indicate that BMP9 may be further explored as a therapeutic factor for these neurons in conditions characterized by their malfunction.

## Materials and Methods

### Animals and Procedures

CD-1 male mice (5 animals per group in each experiment) were used between 40 and 60 days of age. Mice were deeply anesthetized (i.p.) with a mixture of ketamine (90 mg/kg) and xylazine (8 mg/kg) and administered a single injection of prophylactic antibiotic therapy (Ampicillin, 30 mg/kg, i.p.). Their heads were shaved, and the mice were placed in a stereotaxic apparatus. All interventions were performed aseptically on a sterile field (3 M Steri-Drape, C#: 1021). A midsagittal incision (1 cm length) was performed on the scalp, and a subcutaneous tunnel was opened between the shoulder blades, where the pumps were implanted. The periosteum on the surface of the skull was removed by gently rubbing with a sterile Q-tip. Once the bregma had been identified, the tip of the ICV cannula device held in the stereotaxic apparatus was positioned at coordinates bregma −0.5 mm, right-lateral 1 mm. A hole was then opened in the skull (1 mm diameter) and the cannula device was lowered and fixed in place with Loctite-454 adhesive. The tip of the cannula was located in the right lateral ventricle at a depth of 2 mm from the surface of the skull. In addition, for the fimbria-fornix transection, a coronal 2 mm wide slot was opened in the skull on the contra lateral side, and the tip of a 2 mm wide blade was inserted at coordinates: bregma −0.4 mm; left-lateral 0.3 mm; depth 4.0 mm and subsequently removed. At the end of the operation, the incision was closed with silk sutures and the animals were left to recover from the anesthesia and monitored, before they were placed back into their cages. In general, mice were treated with an analgesic (Buprenex), recovered quickly, and were actively feeding within the first 12 hours after the procedure. To determine a dose-response curve to BMP9 (human recombinant, Wyeth Research), mice were implanted with ICV cannulae (Brain Kit #2; Durect Inc.) and osmotic pumps (1007D Alzet pumps; Durect, Inc.), containing 100 µl of 1.6, 16, 32 or 76 ng/µl BMP9, or vehicle (PBS, pH 7.4), and euthanized 6 or 7 days after. The pumping rate was 0.5 µl/h; hence, mice treated with the BMP received 0.8, 8, 16 or 38 ng/h of BMP9, respectively. Once an effective concentration of BMP9 had been established, similar experiments were performed, in which half of the animals received BMP9 at a single concentration of 16 ng/µl (8 ng/h) for 6 days, and half received vehicle. At the time of euthanasia, the pumps were removed and inspected to assure that they had worked properly, and the brains were dissected and fixed for immunohistochemistry, or processed for other assays. All experiments with animals were approved by the Boston University Institutional Animal Care and Use Committee.

### Immunohistochemistry and cell count

Brains were fixed in 20 volumes of PLP fixative (4% paraformaldehyde, 75 mM lysine, 10 mM sodium periodate; pH 7.4) for 1–2 h; they were transferred in fixative to refrigerator (4–8°C) overnight, then cryoprotected in a graded series of 10% and 20% glycerol/2% dimethylsulfoxide, in 0.1 M PBS, pH 7.3 (24 h each). Brains were maintained at 4°C until sectioning. Serial, frozen sections (50 µm, coronal) were cut with a sliding microtome from the anterior frontal pole of to the caudal occipital region. Sections were maintained in PBS/sodium azide in 6-well trays until analysis. For CHAT immunohistochemistry, sections were transferred to Tris-buffered salt solution, containing Tween-20 (TBS-T) and 10% rabbit serum for 1 h at room temperature. Following blocking of sections in serum, they were incubated overnight at room temperature with primary anti-CHAT antibody (Millipore AB144P; 1∶200) and 2% rabbit serum in TBS-T solution. Sections were then washed, blocked with 2% rabbit serum for 1 h, and incubated for 2 h with secondary antibody (Chemicon AQ106P; 1∶1000) in 2% rabbit serum-containing TBS-T. After washing sections in TBS-T, they were transferred to a developing solution containing diaminobenzidine (DAB), sodium imidazole, and hydrogen peroxide. To stop the reaction, sections were then transferred to PBS, mounted on subbed slides and coverslips sealed with Permount.

Brain sections were analyzed on an Olympus B061 microscope (2× and 4× magnification objectives) connected to a megapixel firewire high resolution camera (PixeLink). Serial images, 3 to 4 non-consecutive 100–300 µm apart, from the septal/diagonal band area (region of interest, or ROI) were processed through PixeLink software, stored in a computer, and analyzed with ImageJ software (NIH) and an image-based tool for counting nuclei (Center for Bio-image Informatics, UC Santa Barbara). The ROI was defined by a triangular shape (Fig. 1) that extended, dorso-ventrally, from the apex of the medial septum to an imaginary line connecting the lower limits of the anterior commissures on each hemisphere (Fig. 1, A) and, medio-laterally, from the midline (Fig. 1, D) to the outer limits of the medial septal area (Fig. 1, B and C). The data were analyzed by ANOVA and regression analyses.

### Acetylcholine content analysis

Hippocampi were dissected and collected in methanol, with 1 N formic acid (10∶1 v/v), and immediately homogenized. ACh was extracted from the methanol extract in chloroform and water (1∶2∶1 v/v). The aqueous phase was collected, dried under a vacuum, and reconstituted in water. The content of ACh was determined by HPLC with an enzymatic reactor containing acetylcholinesterase and choline oxidase in series with an electrochemical detector (Bioanalytical Systems, Inc.) based on the method of Potter [44].

### NGF and FGF-2 ELISA

Whole tissue extracts were prepared by adding lysis buffer (50 mM Tris pH 7.5, 150 mM NaCl, 1% Nonidet NP-40, 10% glycerol, 2 mM 4-(2-aminoethyl)- benzenesulfonyl fluoride, 1 µg/ml leupeptin, 2 µg/ml aprotinin, 2 µg/ml pepstatin), followed by gentle sonication, incubation on ice for 15 min, and a brief centrifugation to clear. The supernatant from each sample was diluted 4 times with Dulbecco's PBS and acidified to pH 2.6. After 15 min of incubation at room temperature, the diluted supernatants were neutralized to pH 7.6, aliquoted and frozen for subsequent measurement of NGF and FGF-2 (basic FGF) using ELISA.

The Emax® immunoassay systems (Promega) were used to measure NGF in the samples. Flat-bottom 96 well plates (NUNC) were first coated with solution containing a polyclonal antibody against NGF (the first primary antibody solution) prepared in carbonate coating buffer (100 µL/well, 1∶1000 dilution) and incubated for 16 h at 4°C. Following a wash in TBST (Tris-buffered saline solution containing Tween 20), the coated wells were incubated with block and sample buffer (1×) for 1 h at room temperature and washed again with TBST. Standard control samples for NGF were diluted serially (1∶2) from 250–0 pg/mL and plated in duplicate (100 µL/well). The frozen ELISA samples (described above) were thawed on ice, and every sample plated in duplicate for measurement of NGF. Following a 6 h incubation at room temperature, wells were washed in TBST. Diluted monoclonal antibody against NGF (the second primary antibody solution; 1∶4000) was added to each well and incubated overnight at 4°C. The wells were washed in TBST, incubated with a secondary antibody conjugated to peroxidase for 2.5 h, washed again in TBST, and treated with tetramethyl benzidine (TMB) substrate for 10 min. The chromogen reaction was stopped by adding 100 µL of 1 N hydrochloric acid.

The Quantikine® sandwich ELISA kit (R&D Systems) was used to assay FGF-2 levels in the samples. ELISAs were performed according to manufacture's instructions. Briefly, microplates were pre-coated with the first primary antibody (FGF-2 monoclonal). Assay diluents were added to each well of the microplate (100 µL/well). Standard control samples for FGF were diluted serially (1∶2) from 32–0 pg/ml with its respective calibrator diluent and plated to two columns of wells (100 µL/well) designated for standard curve in every plate. The frozen ELISA samples (described above) were thawed on ice, and every sample plated in duplicate for measurement of each of the factors. Following a 2-h incubation at room temperature, wells were rinsed in wash buffer and treated with an enzyme-linked second primary antibody solution for 2 h. The second primary antibody was a monoclonal FGF-2 antibody conjugated to alkaline phosphatase. The wells were rinsed in wash buffer and a substrate solution was added to the wells and incubated in the dark for 45 min for FGF-2. The color reaction was stopped with 2N sulfuric acid.

The optical density of each well was measured using the Victor3 microplate reader (PerkinElmer Life Sciences). The intensity of color was measured at a wavelength of 450 nm for NGF and at 490 nm for FGF-2. In order to correct for optical imperfections in the plate, readings at 540 nm were subtracted from readings at 450 and 490 nm for NGF and FGF, respectively. The standard curve was used to assess the validity of the protocol and to determine the relative concentrations of NGF. Values in all samples were normalized per gram of tissue assayed, and the average value for each sample was calculated separately before determining the group means.

### Western blot

For Western blot analysis, the lysates were prepared as described in the ELISA methods; however, a portion of the lysate was taken before dilution in Dulbecco's PBS. The extracts were normalized for total protein and subjected to SDS-PAGE. After transfer of protein to an Immoblon P membrane (Millipore), the membrane was blocked with 5% nonfat dry milk in 1× Tris-buffered saline (TBS) containing 0.1% Tween 20 for 2 h and then probed overnight with either anti-NGFR-p75 polyclonal antibody (1∶1000) (Adavanced Targeting Systems); anti-CHAT polyclonal antibody (1∶1000) (Millipore); anti-CHT polyclonal antibody (1∶1000) (Millipore); anti-TrkA polyclonal antibody (1∶1000) (Millipore) or anti-β-actin monoclonal A5441 antibody (1∶5000) (Sigma). The antibody/antigen complexes on the membranes were detected using a peroxidase-conjugated secondary IgG (1∶2000) and visualized using the enhanced chemiluminescence method (Western Lightning, Perkin Elmer) and a Kodak Image Station 440. Digitized images of immunoblots were quantified using Kodak ID software. Protein levels were normalized with β-actin values.

### Data analysis

The statistical analysis of changes in protein levels and ACh content was determined by two-way ANOVA, as appropriate. All post hoc analysis were done using Fisher's least-significant-difference (LSD) test, with p<0.05 deemed as significant. Specific p values are noted in the figure legends. All error bars indicate the SEM.
